# Use of Flavonoids and Cinnamates, the Main Photoprotectors with Natural Origin

**DOI:** 10.1155/2018/5341487

**Published:** 2018-11-28

**Authors:** Alesandra R. Nunes, Ícaro G. P. Vieira, Dinalva B. Queiroz, Antonio Linkoln Alves Borges Leal, Selene Maia Morais, Débora Feitosa Muniz, João Tavares Calixto-Junior, Henrique Douglas Melo Coutinho

**Affiliations:** ^1^Biotechnology Doctorate of the Northeastern Biotechnology Network, State University of Ceará, Av. Dr. Silas Munguba 1700, CEP 60714-903, Campus do Itaperi, Fortaleza, Ceará, Brazil; ^2^Laboratory of Microbiology and Molecular Biology, Department of Biological Chemistry, Regional University of Cariri, Av. Cel. Antonio Luiz, 1161, CEP 63105-000, Pimenta, Crato, Ceará, Brazil

## Abstract

Many pathological problems are initiated by ultraviolet radiation (UVR), such as skin cancer, the most commonly diagnosed cancer worldwide. The UVA (320–400 nm) and UVB (290–320 nm) wavelengths may cause effects such as photoaging, DNA damage, and a series of cellular alterations. The UVA radiation can damage the DNA, oxidize the lipids, and produce dangerous free radicals, which can cause inflammation, modify the gene expression in response to stress, and weaken the skin immune response. With a minor penetration, the UVB radiation is more harmful, being responsible for immediate damage. Ultraviolet radiation light emitted by the sun is considered necessary for the existence of life but cause radiation problems, especially in the skin. The photoprotective activities of plant extracts and isolated composts were evaluated by many reports, as well as the correlation of these compounds with the antioxidant activity. This review presents plant compounds with interest to the cosmetic industry to be used in sunscreens such as flavonoids and cinnamates.

## 1. Introduction

Many pathological problems are initiated by ultraviolet radiation (UVR), such as skin cancer, the most commonly diagnosed cancer worldwide that can cause a high mortality rate when it evolves into a worst type, the melanoma. The UVA (320–400 nm) and UVB (290–320 nm) wavelengths that act in the skin cause effects such as photoaging, photosensitivity (DNA damage), appearance of wrinkles, and a series of cellular alterations involving fibroblasts and melanocytes due to UVA radiation, which possesses a greater power to penetrate the skin. The UVA radiation can damage the DNA, oxidize the lipids, and produce dangerous free radicals, which can cause inflammation, break the cellular communication, modify the gene expression in response to stress, and weaken the skin immune response. But both radiations are linked to skin cancer [1–4]. With a minor penetration, the UVB radiation is more harmful, being responsible for immediate damage, causing burns, skin cancer, and cataracts [5, 6].

Continuous sun exposure not only hinders the skin's ability to repair itself but also continues to break down and debilitate the synthesis of new collagen. UV radiation can also lead to degradation of elastin fibers, causing premature decrease in skin flexibility [7].

Skin cancer is a major health problem worldwide. It is a disease caused by the abnormal and uncontrolled growth of the skin cells. These cells are organized in layers, and the type of cancer will be defined according to the affected layer. It is a public health problem and is the most common in the United States; one in five Americans will develop skin cancer in their lifetime. Currently, between 2 and 3 million nonmelanoma skin cancers and 132000 melanoma skin cancers occur globally each year. In Brazil, 5% of skin cancer cases are melanoma, which has a 75% mortality rate. The most common are the basal cell carcinoma and the squamous cell carcinoma. The cutaneous melanoma is the most aggressive type of skin cancer; it is also rare and deadly [8–12].

All of these effects and new cancer cases (min. 10%) can be prevented or mitigated if people use cosmetic formulations such as sunscreens properly and continuously [1, 13, 14].

The skin is naturally protected from light damage, but when there is excessive exposure to the sun rays, the body may not be able to neutralize them completely, which can lead to many health problems. The use of compounds with antioxidant action could maintain a healthy skin barrier. In addition, they may be useful in photoprotector photostability and may potentially neutralize free radicals induced by UVA radiation [15].

There is of great interest and challenge from the pharmaceutical and cosmetic industries in the development of topical preparations that provide continuous physical, chemical, and biological photoprotection. In addition to that, the damage caused by the synthetic assets has contributed in the development of products whose majority composition is from natural origin, especially vegetable origin, being high quality products with a plurality of benefits [16]. Many costumers' cosmetics are made with natural products as health products. According to Bortolozi [17], Brazil is responsible for 20% of the world consumption of sunscreen, being the world leader.

There is little information about how people in the ancient world protected themselves from the sun. In the 1st century BC, oils, tars, and plant extracts were the various forms used as sunscreens [18].

Rittler, in 1801, discovered the ultraviolet spectrum, and Everard Home, in 1820, discovered the role of the sunlight in skin burns. With that, numerous substances were researched for their ability to absorb this radiation in order to reduce its effects on the skin. Acidified quinine sulfate was the first substance utilized for this purpose by Widmard in 1889, and, in 1891, Hammer incorporated this substance in lotions and ointments, thus creating the first chemical sunscreen in history. Since then, other sunscreens have been appearing, such as esculin, benzyl salicylate, derivatives of *p*-amino benzoic acid, cinnamates, and benzophenone, among others [16].

Only in 1928 did the first sunscreen containing benzyl salicylate and benzyl cinnamate become available in the market in the United States of America. The first patent only came in 1943, with the *p*-amino benzoic acid (PABA). Popularization only occurred in the 1970s, and sunscreens became more effective with the introduction of inorganic titanium dioxide in 1989 and zinc oxide in 1992 [16, 19].

Brassicaceous plants are characterized by a pronounced metabolic flux toward sinapate, produced by the shikimate/phenylpropanoid pathway, which is converted into a broad spectrum of O-ester conjugates. Sinapoylmalate is involved in protecting the leaves against the deleterious effects of UV-B radiation [20].

According to the Skin Cancer Foundation [21], sunscreens help prevent the sun's ultraviolet (UV) radiation from reaching the skin. They protect the skin from UVA and UVB radiations that excessively damage it and increase the risk of skin cancer. They are products consisting of several ingredients and vary in their ability to protect against UVA and UVB.

Sunscreens are used on the skin to minimize or protect from acute and chronic effects of ultraviolet rays, including elimination of free radicals. They are composed of chemical and/or physical sun filters [18, 21].

## 2. Types of Sunscreens and Mechanism of Action

### 2.1. The Inorganic Sunscreens

Inorganic sunscreens reflect and scatter UV and visible radiation, while organic sunscreens absorb UV radiation and then reemit energy as heat or light. These synthetic molecules have limited concentration according to regulation concern. Several natural compounds with UV absorption property have been used to substitute for or to reduce the quantity of synthetic sunscreen agents. In addition to UV absorption property, most natural compounds were found to act as antioxidants and anti-inflammatory and immunomodulatory agents, which provide further protection against the damaging effects of UV radiation exposure. Compounds derived from natural sources have gained considerable attention for use in sunscreen products and have bolstered the market trend toward natural cosmetics. This adds to the importance of there being a wide selection of active molecules in sunscreen formulations [22].

The inorganic sunscreens are metal oxides that generally protect the skin by reflecting the radiation. Zinc oxide and titanium dioxide are the inorganic substances most utilized for photoprotection. They present excellent protection against UV radiation and their advantage is that they are chemically inert and hence do not cause allergic sensitization. These substances are recommended for the manipulation of pediatric photoprotection and for people with sensitive skin. These particles are often visible on the skin, and it becomes an inconvenience for consumers of these cosmetics [5, 14]. However, inorganic oxides such as TiO_2_ and ZnO exhibit their protecting effect only to a small extent by reflection (and scattering), but mostly by absorption, due to their semiconductor band-gaps [23].

The resolution RDC n. 47 [24], from March 16th 2006, established a maximum concentration of 25% of these inorganic sunscreens in the formulation of products.

### 2.2. Chemical Filters

Chemical sunscreens are organic compounds. Usually, these compounds protect the skin by absorbing the radiation. Currently, there are organic sunscreens in the market that not only absorb 95% of the (290–320 nm) wavelengths, protecting against erythema and skin wrinkling, but also reflect UV radiation [5, 14]. Many of these molecules are aromatic compounds that have carboxyl groups and, generally, they present an electron-donating group, such as amine or a methoxyl group, in the ortho or para positions [25].

Different studies show that, despite the fact that these sunscreens should be used on the skin surface, absorption, metabolization, accumulation, and elimination of some organic filters by the organism may occur. This percutaneous absorption can result in harmful processes to the health, such as contact dermatitis, mutations, cancer, estrogenic activity, or photosensitivity reactions [5, 26].

Thus, the photoprotection from naturally occurring substances has gained considerable attention in recent years due to their wide range of biological activities. The development of sunscreens, which include natural products with a broad spectrum of efficacy and low toxicity, is of great interest to the cosmetic industry [16].

The main synthetic organic filters are *p*-aminobenzoic acid (PABA), cinnamates (*p-*octyl-methoxycinnamate), salicylates, benzimidazoles (2-phenylbenzimidazole acid, derivatives 5-sulfonic benzylidene), camphor, and benzophenone [27]. However, recent researches appointed the triazine and benzoltriazole derivatives with biggest potential and have gained importance worldwide [28].

In 1928, in the USA, one of the first sunscreens had benzyl cinnamate in its composition, but its use was limited. Among the currently most used UV chemical filters is the octyl methoxicinnamate [26]. Commercial compounds have several problems, such as low photostability and endocrine disruption properties. Scientific studies show that chemical filters are capable of altering the hypothalamic-pituitary-thyroid (HPT) axis, also affecting the homeostasis of the reproductive axis, as well as some development parameters [29, 30].

Currently, *in vitro* and *in vivo* techniques are used in the determination of the sun protection factor (SPF) and the UVA protection factor (APF) of sunscreens. Among the determination techniques of these *in vitro* factors recommended by the FDA (Food and Drug Administration Agency) and COLIPA (European Cosmetic, Toiletry, and Perfumery Association) the reflectance spectrophotometer with integrating sphere stands out. This technique is mainly applied with opaque materials, such as the cosmetic products used as sunscreens [31, 32].

The *in vitro* techniques to evaluate products as sunscreens present advantages: the practicality to be employed routinely in quality control to assure that each batch of sunscreen is adequate for use; the speed; the affordable cost and reproducibility that guarantee an effective preliminary evaluation of the natural products in the Brazilian flora for future use in the cosmetic industry as photoprotectors [33, 34].

## 3. Determination of Photoprotection Activity *In Vitro*

The *in vitro* methods are, in general, of two types: methods that involve the measurement of absorption or the transmission of UV radiation through sunscreen product films in quartz plates or biomembranes and methods in which the absorption characteristics of the sunscreen agents are determined based on spectrophotometric analysis of dilute solutions. The efficacy of a sunscreen is usually expressed by the sun protection factor (SPF), which is defined as the UV energy required to produce a minimal erythemal dose (MED) in the protected skin divided by the UV energy required to produce an MED in unprotected skin. The minimal erythemal dose (MED) is defined as the lowest time interval or dosage of UV light irradiation sufficient to produce a minimal, perceptible erythema on unprotected skin. To this determination, oils of various manufacturers can be purchased from local pharmacies. The solubility of oils is determined in different ratios of ethanol and distilled water. It is reported that a maximum of 50% of ethanol could be used in cosmetics. Hence, solubility of oils is detected taking 10% to 50% of ethanol in distilled water. The maximum solubility is observed in 40% ethanol and 60% distilled water solution [35].

### 3.1. Wavelength Determination and Plant Extracts Absorbance

This analysis is performed in a spectrophotometer in order to determine maximum wavelength (λmax) and maximum absorbance (Amax) of plant extracts dissolved in solvents that are not supposed to absorb ultraviolet radiation. The most widely used are water, 95% ethanol, and hexane. A scan is carried out between the lengths 260–400 nm to verify absorption in the UVA (320–400) and UVB (290–320) regions [36, 37].

### 3.2. *In Vitro* Evaluation of Sun Protection Factor by Mansur Method

It is a method to determine the SPF by spectrophotometric reading of diluted solutions of samples and further mathematical treatment by determining the transmittance or absorbance (*T* = 10–Abs) in the wavelenghts 290–320 nm, with 5 nm breaks, in quartz cuvettes. The solvents used in this technique are usually methanol, isopropanol, or ethanol. The samples are dissolved at a concentration of (0.2 mg/mL) and analyzed in triplicate. The absorbances obtained are added to the equation shown below, which determines the sun protection factor [36, 38–40].

Table 1 shows the sun protection factor (SPF), according to Mansur's method [39], of several plant extracts, where *Aniba canelilla*, *Calendula officinalis*, and *Euphorbia tirucalli* showed the best activities.

(1) $$\begin{array}{c} \text{SFP}=\text{CF}\times \overset{320\text{ mm}}{\underset{290\text{ mm}}{\sum }}\text{EE}\lambda \times \text{I}\lambda \times \text{Abs}\lambda , \end{array}$$

where CF = correction factor = 10, EE*λ* = erythematogenic effect of radiation wavelength (*λ*), I*λ* = sunlight intensity at a wavelength (*λ*), and Abs*λ* = spectrophotometric determination of absorbance of the formulation in solution at a wavelength (*λ*).

## 4. Antioxidant Effect and Its Association with Photoprotective Effect of Natural Products

**Figure 1:**
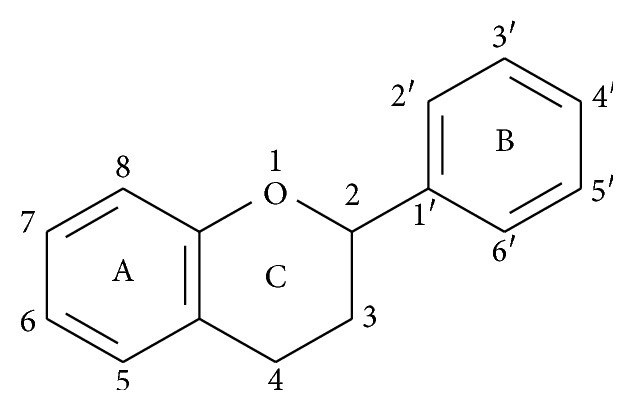
Basic skeleton structure of flavonoids.

The plant kingdom produces secondary metabolites that are synthesized to develop protective functions against hostile environments, such as oxidation by solar radiation. These metabolites are constituted by a large number of compounds, and among these, we highlight polyphenols, such as flavonoids and phenolic acids, whose synthesis does not occur in humans. Flavonoids have a basic nucleus in common that can be subdivided into different subgroups depending on the carbon of the C ring on which the B ring is attached and the degree of unsaturation and oxidation of the C ring. They are called isoflavones, neoflavonoids, and other subgroups (Figure 1), having the ability to absorb ultraviolet radiation due to the presence of chromophores in their structure. Rutin (3-*O*-rutinoside-quercetin), part of the flavonols class, can then be used as sunscreen in cosmetic formulations [41–44].

Arct and collaborators; Valenta, Nowack, and Bernkop-Schnürch; and Saija et al. studied the penetration and permeation of flavonoids by in vitro diffusion cell methodologies [44–46]. Bobin et al. evaluated the permeation of rutin, catechin and quercetin in the presence of adjuvants hydrophilic (wetting) and their influence on the permeation of the flavonoids through an artificial membrane [45]. According to the experimental results obtained, these acted as inhibitors of the permeation of flavonoids under study, however, at different levels of inhibition [49].

Flavonoids, tannins, alkaloids, anthraquinones, and polyphenols are secondary metabolites present in plants and considered natural sunscreens, since they have the property of absorbing light in the ultraviolet region [5, 42]. Flavonoids have three different actions: capability of absorbing ultraviolet lights, antioxidant properties, and modulating several signaling pathways. The ultraviolet absorption spectrum of a flavonoid generally shows two maximum peaks of absorption, one between 240 and 280 nm and the other at 300–550 nm, which shows the possibility of using these extracts as sunscreens in both UVB and UVA photoprotection preparations [3, 43].

The synergistic effect has been shown for the combination of 0.1% rutin and 6.0% benzophenone, which caused a SPF increase from 24.3 ± 1.53 to 33.3 ± 2.89 when the flavonoid was included [45].

According to a study by Filho et al. [46], rutin and quercetin, potent antioxidants, incorporated a water-in-oil emulsion, used in concentrations of 10%, individually associated with titanium dioxide and zinc oxide, obtained synergistic effect with a SPF value about 30.

The hydroalcoholic extract of the leaves of the plant species *Arrabidaea chica*, which belongs to the bignoniaceae family, presented in spectrophotometric analysis in the UVB region (283 nm and UVA region 331 nm) peaks in the concentration 0.10 mg/mL [47].

Propolis is a complex mixture, which includes the presence of flavonoids (flavones, flavonones, and flavonols), aromatic acids and esters, aldehydes, ketones, terpenes, steroids, amino acids, phenylpropanoid, fatty acids, hydrocarbons, and many other compounds in smaller proportions. Its chemical composition varies according to the collection site, due to the vegetation. This complexity gives propolis numerous pharmacological properties, among them is the ability to absorb UV rays. Several studies have been done concerning this property: the synergistic effect of the SPF of glycolic extracts and red and green propolis ethanol with UVA/UVB water-soluble sunscreens (association of 2-phenylbenzimidazole-5-sulfonic acid–Eusolex 232®–and 2-hydroxy-4-methoxybenzophenone-5-sulfonic acid, 1,2-propanediol and demineralized water) [48]. Propolis dry extract in 3, 10, and 40% concentrations presented SPF values ranging from 1 to 12 and a synergistic effect in combination with octyl methoxycinnamate [38].

Ethanol extracts of *Aniba canelilla* (Kunth) leaves, according to phytochemical study, revealed the presence of flavonoids and showed to be promising for the use in products for sun protection. However, other studies should be conducted so that the constituents responsible for the effect are characterized [49].

Pycnogenol, the name given to the extract derived from the pine bark of a tree known as *Pinus pinaster*, contains a number of phenolic and polyphenolic flavonoids. Acute and chronic effects of UV radiation exposure were reduced significantly in experimental animals after topical application of pycnogenol. The red clover (*Trifolium pratense*) presents a high content of antioxidant flavonoids like genistein and daidzein. Flavonoids such as genistein (4′,5,7- trihydroxyisoflavone, soybean isoflavone) are potent antioxidants and effectively block UVB-induced skin burns in humans. Silymarin, a mixture of three flavonoids (silybin, silydianin, and silychristin), equol, a metabolite of daidzein produced exclusively by the action of intestinal microflora of mammals, quercetin, and apigenin are alsot flavonoids that have excellent photoprotective properties, but present problems such as chemical instability and should be further studied. In a study using silymarin (SM) and silybin (SB), the photoactive effect by UVA stimulated the damage against primary human dermal fibroblasts. It results in a reduction in UVA-stimulated ROS generation and SSB production, as well as in the prevention of GSH depletion, a decrease in the activation of caspase-3 and protein level of MMP-1. This data showed that both SM and SB are nonphototoxic and have UVA-photoprotective potential and could be useful agents for UV-protective dermatological preparations [50–52].

**Figure 2:**
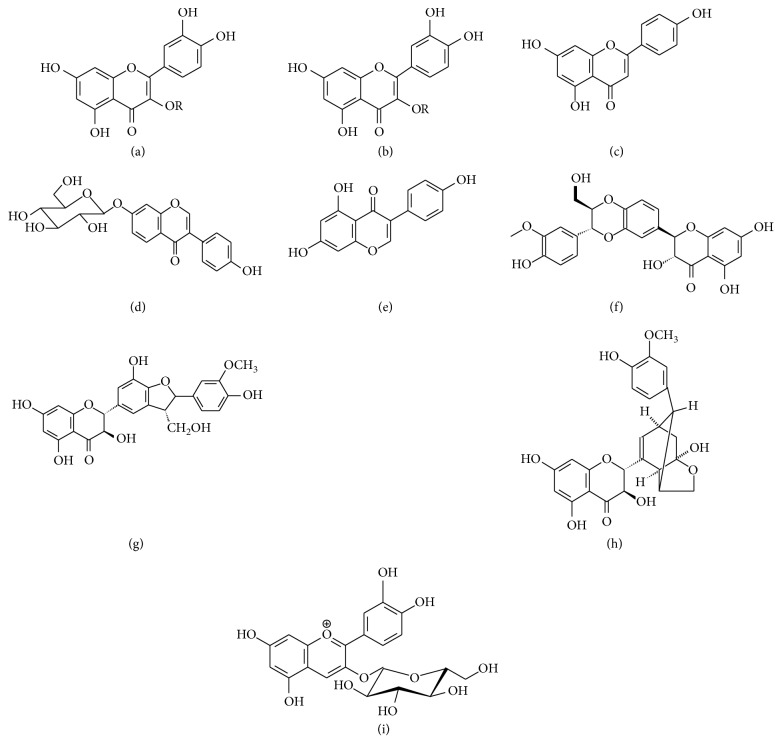
Some photoprotector flavonoids. (a) Quercetin, R= H. (b) Rutin, R= rutinose rutin. (c) Apigenin/cosmosiin. (d) Daidzein. (e) Genistein. (f) Silibinin. (g) Silychristin. (h) Sylidianin. (i) Cyanidin-3-glucoside.

The most common anthocyanin found in nature, cyanidin-3-glucoside, has photoprotection against UVA and UVB over the human keratinocytes; some adverse effects of UVB exposure are inhibited when the cells are pretreated with cyanidin-3-glucoside [5, 53].  Figure 2 shows the chemical structures of some flavonoids with photoprotective activity.

Witch hazel (*Hamamelis virginiana* L.), chamomile (*Matricaria recutita* L.), conker tree (*Aesculus hippocastanum* L.), cascara buckthorn (*Rhamnus purshiana* DC), and cinnamon (*Cinnamomum zeylanicum* Nees) have varied chemical compositions. The following compounds are present: flavonoids, tannins, anthraquinone derivatives, and cinnamates. All extracts with 10% of the above-mentioned plants had a good absorption in the UV region, and there was a significant increase in this absorption when individually mixed in a octyl methoxycinnamate solution, potentiating this effect. They showed high stability regarding contamination and degradation [54, 55].

The *Carica papaya* extract, rich in flavonoids and phenolic compounds, displays antioxidant activity and has demonstrated stability when exposed to UVB radiation. The SPF by the Mansur method was 16.047 ± 0.05; adding 1.42 mg of *C. papaya* extract in formulation cream base, the SPF was 2.9 ± 0.01 [56].

A study done [54] with crude extracts of *Guazuma ulmifolia* (West Indian elm), *Maytenus ilicifolia* (espinheira-santa), *Stryphnodendron adstringens* (barbatimão), and *Trichilia catigua* (catuaba) that present total polyphenols in their composition were not considered as potential photoprotectors in the evaluated conditions. Samples were prepared in a protective formula with a concentration of 10 mg/g of total polyphenols of each extract. Only one sample, containing the *S. adstringens* extract, showed a slight increase of SPF, which, however, was not statistically significant.

The *Arrabidaea chica* (Bignoniaceae) contains flavonoids, anthocyanins, and tannins. A formulation with 5% of crude extract in a nonionic cream presented a good absorption both in the UVA and UVB regions, stability, and no toxicity in the animals tested [55].

The leaf ethanol and hydroacetone extracts of *Bauhinia microstachya* var*. massambabensis* were incorporated in sunscreen creamy lotions, generating formulations considered satisfactory in efficacy and safety tests. The formulations showed stability, safety, and no toxicity. Although they do not have a photoprotective capacity, their presence, regardless of the concentration used, increased the SPF of formulations with other synthetic chemicals [57].

Cinnamic acid derivatives are the best candidates for UVB filters in the (310–325 nm) wavelength. A formulation containing a mixture of caffeic acid with caffeic acid phenylethyl ester, or dimethyl caffeic acid, which are polyphenols with photoprotective activity, could provide significant protection values in both the UVA and UVB regions [46, 58].

**Figure 3:**
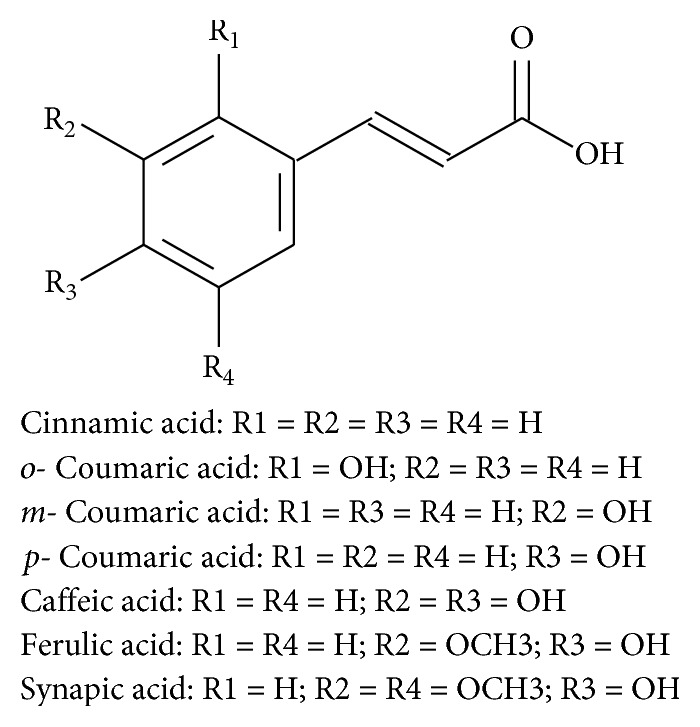
Chemical structure of major cinnamic acids. Cinnamic acid: R1 = R2 = R3 = R4 = H. *o*-Coumaric acid: R1 = OH; R2 = R3 = R4 = H. *m-*Coumaric acid: R1 = R3 = R4 = H; R2 = OH. *p-*Coumaric acid: R1 = R2 = R4 = H; R3 = OH. Caffeic acid: R1 = R4 = H; R2 = R3 = OH. Ferulic acid: R1 = R4 = H; R2 = OCH3; R3 = OH. Synapic acid: R1 = H; R2 = R4 = OCH3; R3 = OH.

One of the plants with great potential for use as a source of products with photoprotective activity is the carnauba (*Copernicia cerifera*), a Brazilian palm tree that has leaves covered by a waxy coating. This material, after being extracted from the leaves, becomes a raw material for the production of carnauba wax, the most important vegetable wax from the economic aspect with various applications [59, 60]. This product is of great commercial interest for use in organic filters due to its high content of cinnamate derivatives such as aliphatic diesters of *p*-methoxicinnamic acid or hydroxy cinnamic acid. The cinnamates are present in many sunscreens [61].  Figure 3 shows the structure of the major natural cinnamic acids, which are present in plants either free or in the form of esters.

In a study with encapsulation of titanium dioxide with carnauba wax, there was a significant increase in the value of the sun protection factor of this oxide. The authors consider this increase as proof of a previous study that showed that increasing the viscosity of cosmetic preparations containing titanium dioxide was directly related to the increase in SPF [62]. However, a study conducted by Freitas et al. [63] isolated a fraction rich in cinnamic esters from the carnauba wax and showed an excellent absorption in this UV region and also antioxidant activity, showing a promising future for cosmetic formulations such as sunscreens, since it would be a stable organic compound obtained from a natural source.

## 5. Conclusion

Flavonoids and cinnamates present in many plant extracts have essential characteristics to be sunscreens, such as absorption of ultraviolet rays, as well as antioxidant activity, usefulness in cosmetics, action against free radicals, and controlling the early ageing caused by various external factors.

There was a synergistic action in several combinations of these compounds or extracts mixed with synthetic filters, thus increasing the sun protection factor (SPF), as well as stabilizing certain formulations. The stability is also something that must be investigated, as well as the toxicity of some extracts, and if it can cause allergic problems and increased costs [9, 10, 11].

### 5.1. Future Perspective

It is necessary that further research is carried out to increase the spectrum of products from plants that are recognized as potential photoprotectors. In addition, it is important to search for new methodologies to be addressed to this finality.

## Figures and Tables

**Table 1:** Results for some plant extracts for photoprotective activity.

Scientific name	Popular name	Family	Preparation	SPF (*µ*g·mL^−1^)	Reference
*Ginkgo biloba* L.	Ginkgo	Ginkgoaceae	Ethanol extract	0.82	Pinto et al., n.d. [61]
*Hamamelis virginiana* L.	Witch hazel	Hamamelidaceae	Ethanol extract	0.68	
*Matricaria chamomilla* L.	Chamomile	Asteraceae	Ethanol extract	0.56	
*Pereskia aculeata* Mill	Barbados gooseberry	Cactaceae	Ethanol extract of the leaves	0.69	
*Aniba canellila* (Kunth) Mez	Preciosa	Lauraceae	Essential oils from leaves	14.08	Fonseca Jr. et al. [62]
*Malpighia glabra* L	Acerola	Malpighiaceae	Lotion with 10% of dry fruit extract	0.14	Souza et al. [24]
*Bromelia laciniosa* Mart. ex Schult. and Schult.F	Macambira	Bromeliaceae	Leaf ethanol extract	7.22	Oliveira et al. [63]
*Tabebuia aura* (Silva Manso) Benth. and Hook. f. ex S. Moore	Caribbean trumpet tree	Bignoniaceae	Lotion with ethanol extract	0.38	Violante et al. [41]
*Calendula officinalis* L.	Pot marigold	Asteraceae	Lotion with 5% of the flowers oil	14.84	Ramos et al., 2010 [39]
*Euphorbiatirucali* L.	Aveloz	Euphorbiaceae	Aerial parts ethanol extract	19.82	Ramos et al., 2010 [39]
*Byrsonima sericea* DC	Murici	Malpighiaceae	Leaf ethanol extract	1.36	Ramos et al., 2010 [39]
*Byrsonima sericea* DC	Murici	Malpighiaceae	Leaf aqueous extract	0.68	

## References

[1] Polonini H. C., Lima L. L., Gonçalves K. M., Carmo A. M. R., Silva A. D., Raposo N. R. B. (2013). Photoprotective activity of resveratrol analogues. *Bioorganic and Medicinal Chemistry*.

[2] Diepgen T. L., Fartasch M., Drexler H., Schmitt J. (2012). Occupational skin cancer induced by ultraviolet radiation and its prevention. *British Journal of Dermatology*.

[3] Kale S., Ghoge P., Ansari A., Waje A., Sonawane A. (2010). Formulation and in-vitro determination of sun protection factor of *Nigella sativa* Linn. seed oil sunscreen cream. *International Journal of PharmTech Research*.

[4] Araujo T. S., Souza S. O. (2008). Protetores solares e os efeitos da radiação ultravioleta. *Scientia Plena*.

[5] Saewan N., Jimtaisong A. (2013). Photoprotection of natural flavonoids. *Journal of Applied Pharmaceutical Science*.

[6] Velasco M. V. R., Balogh T. S., Pedriali C. A. (2008). Associação da rutina com p-metoxicinamato de octila e benzofenona-3: avaliação in vitro da eficácia fotoprotetora por espectrofotometria de refletância. *Latin American Journal of Pharmacy*.

[7] Dayan N. (2008). *Skin Aging Handbook: An Integrated Approach to Biochemistry and Product Development*.

[8] Aziz S. W., Aziz M. H. (2018). Protective molecular mechanisms of Resveratrol in UVR induced skin carcinogenesis. *Photodermatology, Photoimmunology and Photomedicine*.

[9] World Health Organization *Skin Cancers. How Common is Skin Cancer?*.

[10] Bertemes P. F., Imai F. A. Comparison between solar radiation and skin cancer in South Brazil.

[11] Sociedade Brasileira de Dermatologia (SBD) *O Que é o Câncer de Pele*.

[12] Nascimento L. F., Santos E. P., Aguiar A. P. (2014). Fotoprotetores orgânicos: pesquisa, inovação e a importância da síntese orgânica. *Revista Virial de Quimica*.

[13] Monteiro E. O. (2010). Filtros solares e fotoproteção. *Revista Brasileira de Medicina*.

[14] Flor J., Davolos M. R., Correa M. A. (2007). Protetores solares. *Quimica Nova*.

[15] Stiefel C., Schwack W. (2014). Reactivity of cosmetic UV filters towards skin proteins: model studies with Boc-lysine, Boc-Gly-Phe-Gly-Lys-OH, BSA and gelatin. *International Journal of Cosmetic Science*.

[16] Urbach F. (2001). The historical aspects of sunscreens. *Journal of Photochemistry and Photobiology B: Biology*.

[17] Kostyuk V. A., Potapovich A. I., Albuhaydar A. R., Mayer W., De Luca C., Korkina L. G. (2017). Natural substances for prevention of skin photo-ageing: screening systems in the development of sunscreen and rejuvenation cosmetics. *Rejuvenation Research*.

[18] Bortolozi T. *Higiene Pessoal, Perfumarias e Cosméticos*.

[19] Schalka S., Dos Reis V. M. S. (2011). Fator de proteção solar: significado e controvérsia. *Anais Brasileiro de Dermatologia*.

[20] Milkowski C., Strack D. (2010). Sinapate esters in brassicaceous plants: biochemistry, molecular biology, evolution and metabolic engineering. *Planta*.

[21] Skin Cancer Foundation *Sunscreens*.

[22] Saewan N., Jimtaisong A. (2015). Natural products as photoprotection. *Journal of Cosmetic Dermatology*.

[23] Hughes M. C. B., Williams G. M., Baker P., Green A. C. (2013). Sunscreen and prevention of skin aging: a randomized trial. *Annals of International Medicine*.

[24] Agência Nacional de Vigilância Sanitária (2016). *Resolução RDC n. 47 de 16 de março de 2006*.

[25] Souza F. P., Campos G. R., Packer J. F. (2013). Determinação da atividade fotoprotetora e antioxidante em emulsões contendo extrato de *Malpighia glabra* L.–Acerola. *Revista de Ciências Farmacêuticas Básica e Aplicada*.

[26] Chisvert A., González Z. L., Tarazona I., Salvador A., Giokas D. (2012). An overview of the analytical methods for the determination of organic ultraviolet filters in biological fluids and tissues. *Analytica Chimica Acta*.

[27] Baillo V. P., Lima A. C. (2012). Nanotecnologia aplicada à fotoproteção. *Revista Brasileira de Farmácia*.

[28] Santos B. A. M. C., P Silva A. C., Bello M. L. (2018). Molecular modeling for the investigation of UV absorbers for sunscreens: triazine and benzotriazole derivatives. *Journal of Photochemistry and photobilogy A Chemistry*.

[29] Losantos R., Ardoiz I. F., Aguilera J. (2017). Rational design and synthesis of efficient sunscreens to boost the solar protection factor. *Angewandte International Edition*.

[30] Garcia E. B., Machado S. C., Ferraris F. K. (2015). Contaminação ambiental e da cadeia alimentar com filtros solares: um potencial risco à saúde humana. *Revista Analytic*.

[31] Nishikawa D. O., Peres D. A., Oliveira C. A. (2013). Estabilidade e eficácia de fotoprotetores contendo filtros inorgânicos e quercetina, Biopharmaceutical Sciences. *Ciências Biofarmacêuticas*.

[32] Velasco M. V. R., Balogh T. S., Pedriali C. A. (2011). Novas metodologias analíticas para avaliação da eficácia fotoprotetora (in vitro): revisão. *Revista de Ciências Farmacêuticas Básica e Aplicada*.

[33] Roca L. M., Moreira S. C. L. M., Moreira A. (2011). Avaliação laboratorial do fator de proteção solar (FPS) em protetores utilizados por portadores de albinismo na Bahia. *Revista de Ciencias Medica e Biologica*.

[34] Santos E. P., Freitas Z. M., Souza K. R., Garcia S. (1999). In vitro and in vivo determinations of sun protection factors of sunscreen lotions with octylmethoxycinnamate. *International Journal of Cosmetic Science*.

[35] Kaur C. D., Saraf S. (2010). In vitro sun protection factor determination of herbal oils used in cosmetics. *Pharmacognosy Research*.

[36] Dengo B. L., Ferreira J. R. N. (2017). Avaliação in vitro do potencial fotoprotetor do extrato do bagaço da uva isabel (*Vitis labrusca* l). *Evidence*.

[37] Violante I. M. P., Souza I. M., Venturini C. L., Ramalho A. F. S., Santos R. A. N., Ferrari M. (2009). Avaliação in vitro da atividade fotoprotetora de extratos vegetais do cerrado de Mato Grosso. *Revista Brasileira de Farmacognosia*.

[38] Velasco M. V. R., Sarruf F. D., Oliveira C. A. (2012). Influência de substâncias bioativas na estabilidade fisico-química e funcional de emulsões fotoprotetoras. *Biomedical and Biopharmaceutical Research*.

[39] Ramos M. F. S., Santos E. P., Ortiz G. M. D. (2010). Avaliação da atividade antisolar e estudos preliminares de fotodegradação da própolis. *Revista Fitos*.

[40] Mansur J. S., Breder M. N. R., Mansur M. C. A., Azulay R. D. (1986). Correlação entre a determinação do fator de proteção solar em seres humanos e por espectrofotometria. *Anais Brasileiro de Dermatologia*.

[41] Panche A. N., Diwan A. D., Chandra S. R. (2016). Flavonoids: an overview. *Journal of Nutritional Science*.

[42] Bizari M. (2013). Desenvolvimento de filtros solares a partir de complexos metálicos de Zn2+ e/ou Ce3+ com quercetina para protetores solares, Química IDE.

[43] Violante I. M. P., Souza I. M., Venturini C., Ramalho A. F. S., Santos R. A. N., Ferrari M. (2008). Estudo preliminar da atividade fotoprotetora in vitro de extratos vegetais do cerrado de Mato Grosso. *Revista Brasileira de Farmácia*.

[44] Lopes R., Oliveira T. T., Nagem T. J., Pinto A. D. S. (2000). Farmacologia de flavonoides controle hiperlipidêmico em animais experimentais. *Biotecnologia Ciêncencia e Desenvolvimeto*.

[45] Bobin M. F., Raymond M., Martini M. C. (1995). Propriedades de absorção UVA/UVB de produtos naturais. *Cosmetics & Toiletries*.

[46] Filho J. M. T. A., Sampaio P. A., Pereira E. C. V. (2016). Flavonoids as photoprotective agents: a systematic review. *Journal of Medicinal Plants Research*.

[47] Choquenet B., Couteau C., Paparis E., Coiffard L. J. M. (2008). Quercetin and rutin as potential sunscreen agents: determination of efficacy by an in vitro method. *Journal of Natural Products*.

[48] Montanha M. C., Ribeiro B. M., Nakamura T. U., Filho B. P. D., Nakamura C. V. (2010). *Padronização de extratos obtidos das folhas da Arrabidaea chica para a produção de um fotoprotetor solar, Encontro Anual de Iniciação Científica: anais dos 19*.

[49] Baby A. R., Filho C. A. H., Sarruf F. D. (2008). Estabilidade e estudo de penetração cutânea in vitro da rutina veiculada em uma emulsão cosmética através de um modelo de biomembrana alternativo. *Revista Brasileira de Ciências Farmacêuticas*.

[50] Nascimento C. S., Nunes L. C. C., Lima Á. A. N., Júnior S. G., Neto P. J. R. (2009). Incremento do FPS em formulação de protetor solar utilizando extratos de própolis verde e vermelha. *Revista Brasileira de Farmácia*.

[51] Júnior E. Q. F., Castilho R. B., Silva G. F. (2015). Avaliação do potencial fotoprotetor dos óleos essenciais e extratos etanólicos de *Aniba canelilla* (HBK). *Blucher Chemical Engineering Proceedings*.

[52] Svobodová E., Gabrielová L., Michaelides P., Kosina A., Ryšavá J., Ulrichová B. (2018). UVA-photoprotective potential of silymarin and silybin. *Archives of Dermatological Research*.

[53] Gilaberte Y., González S. (2010). ACTAS dermo-sifiliográficas update on photoprotection. *Actas Dermo-Sifiliográficas English*.

[54] Munhoz V. M., Lonni A. A. S. G., Mello J. C. P., Lopes G. C. (2012). Avaliação do fator de proteção solar em fotoprotetores acrescidos com extratos da flora brasileira ricos em substâncias fenólicas. *Revista de Ciências Farmacêuticas Básica e Aplicada*.

[55] Ramos M., Santos E. (1996). Preliminary studies towards utilization of various plant extracts as antisolar agents. *International Journal of Cosmetic Science*.

[56] Shenekar P. N., Ukirade P. S., Salunkhe S. D. (2014). In vitro evaluation of sun protection factor of fruit extract of *Carica papaya* L. as a lotion formulation. *European Journal of Experimental Biology*.

[57] Siraichi J. T. G., Pedrochi F., Natali M. R. M. (2013). Ultraviolet (UVB and UVA) photoprotector activity and percutaneous penetration of extracts obtained from *Arrabidaea chica*. *Applied Spectroscopy*.

[58] Mansur M. C. P. P. R. Estudo preliminar das atividades fotoprotetora e antioxidante dos extratos das folhas de Bauhinia microstachya var. massambabensis Vaz numa formulação antissolar.

[59] Agati G., Tattini M. (2010). Multiple functional roles of flavonoids in photoprotection. *New Phytologist*.

[60] Carvalho F. P. A., Gomes J. M. A. (2008). Eco-eficiência na produção da cera de carnaúba município de Campo Maior, Piauí. *Revista de Economia Socioogia Rural*.

[61] Wolfmeier U., Hans-Jurgen A. (2005). *Ullmann’s Encyclopedia of Industrial Chemistry*.

[62] Hernández J. R. V., Goymann C. C. M. (2006). Sun protection enhancement of titanium dioxide crystals by the use of carnauba wax nanoparticles: the synergistic interaction between organic and inorganic sunscreens at nanoscale. *International Journal of Pharmaceutics*.

[63] Freitas C. A. S., Vieira I. G. P., Sousa P. H. M., Muniz C. R., Gonzaga M. L. C., Guedes M. I. F. (2016). Carnauba wax p-methoxycinnamic diesters: characterisation, antioxidant activity and simulated gastrointestinal digestion followed by in vitro bioaccessibility. *Food Chemistry*.

